# Supplementary material for: Immune profiling-based targeting of pathogenic T cells with ustekinumab in ANCA-associated glomerulonephritis
Source: Nat Commun. 2024 Sep 19;15:8220. doi: 10.1038/s41467-024-52525-w (PMC11413367; doi:10.1038/s41467-024-52525-w)
Supplement: Supplementary file 1 — Supplementary Information [file 41467_2024_52525_MOESM1_ESM.pdf]

Immune profiling-based targeting of pathogenic T cells with ustekinumab in ANCA-associated glomerulonephritis

Jonas Engesser[1,2#], Robin Khatri[2,3#], Darius P. Schaub[2,3#], Yu Zhao[1,2,3], Hans-Joachim Paust[1,2], Zeba Sultana[1,2,3], Nariaki Asada[1,2], Jan-Hendrik Riedel[1,2], Varshi Sivayoganathan[1,2], Anett Peters[1], Anna Kaffke[1], Saskia-Larissa Jauch-Speer[1], Thiago Goldbeck-Strieder[1], Victor G. Puelles[1,4], Ulrich O. Wenzel[1], Oliver M. Steinmetz[1], Elion Hoxha[1,4], Jan-Eric Turner[1,2], Hans-Willi Mittrücker[2,5], Thorsten Wiech[4,6], Tobias B. Huber[1,2,4], Stefan Bonn[2,3,4*], Christian F. Krebs[1,2,4*], Ulf Panzer[1,2,4*]

Affiliations:
[1]III. Department of Medicine, University Medical Center Hamburg-Eppendorf; Hamburg 20251, Germany
[2]Hamburg Center for Translational Immunology, University Medical Center Hamburg-Eppendorf; Hamburg 20251, Germany
[3]Institute of Medical Systems Biology, Center for Biomedical AI, Center for Molecular Neurobiology Hamburg; Hamburg 20251, Germany
[4]Hamburg Center for Kidney Health (HCKH), University Medical Center Hamburg-Eppendorf, Hamburg 20251, Germany
[5]Institute for Immunology, University Medical Center Hamburg-Eppendorf, Hamburg 20251, Germany
[6]Institute of Pathology, Division of Nephropathology, University Medical Center Hamburg-Eppendorf; Hamburg 20251, Germany

[#]These authors contributed equally: Jonas Engesser, Robin Khatri and Darius P. Schaub
*These authors jointly supervised this work: Stefan Bonn, Christian F. Krebs and Ulf Panzer

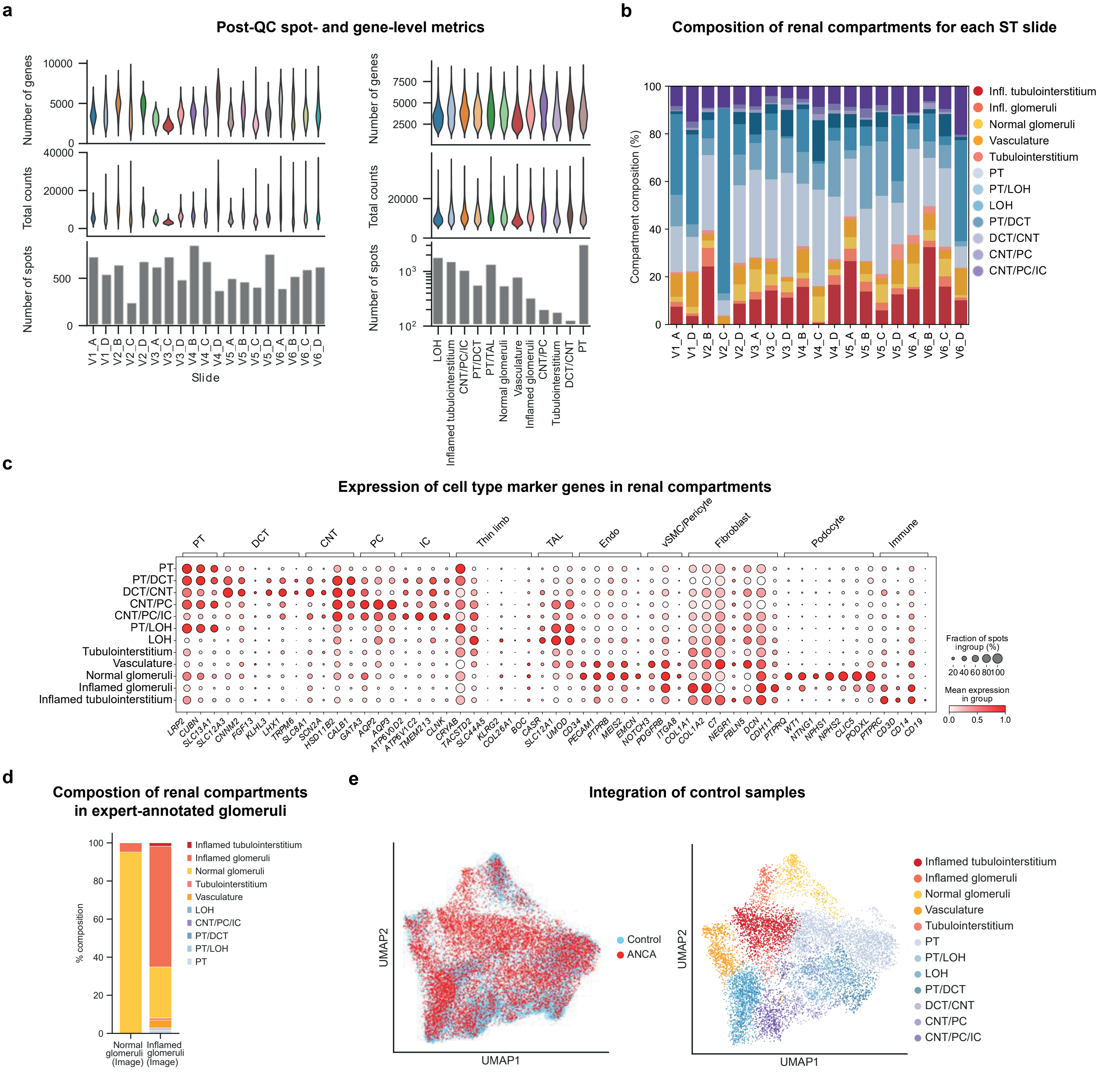

Supplementary Figure 1: Overview of spatial transcriptomics data from the exploratory group.
a Post-quality control (QC) distributions of number of genes, total UMI counts per spot, and number of spots per Visium ST slide (left) and renal compartment (right). **b** Barplots showing composition of each ST slide. **c** Dot plot showing the expression of marker genes across the renal compartments. **d** Barplots showing the distribution of gene-expression-based annotations compared to expert-based image annotations of normal and inflamed glomerular compartments (x-axis). **e** Joint UMAP-embedding of control and ANCA-GN exploratory group showing condition and renal compartments after the integration of ST slides containing control samples (8 slides and 21,420 spots).
LOH, loop of Henle. CNT, connecting tubules. PC, principal cells. IC, intercalated cells. PT, proximal tubules. DCT, distal convoluted tubules. TAL, thick ascending loop of Henle. vSMC, vascular smooth muscle cells.

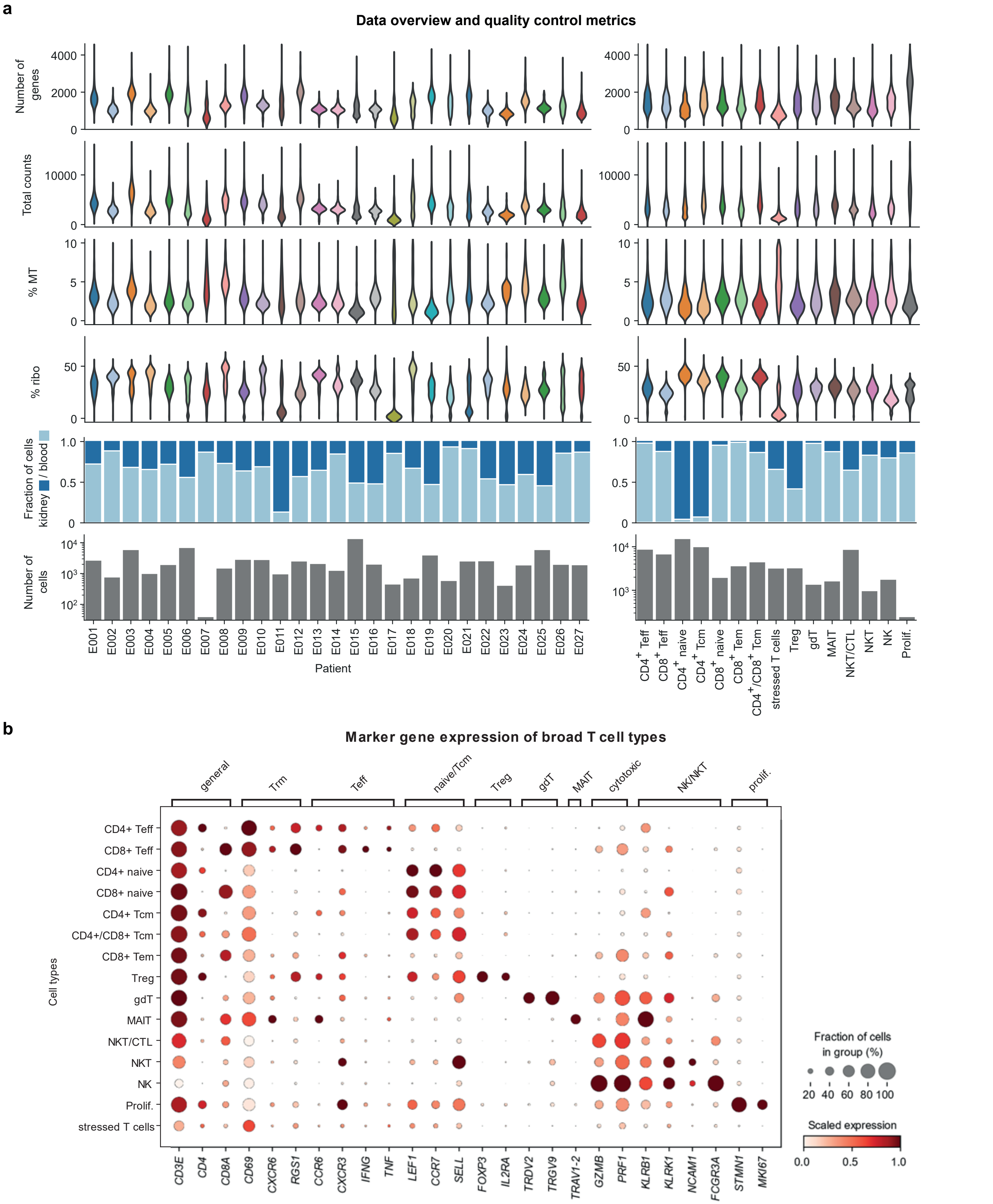

Supplementary Figure 2: Exploratory cohort single cell T cell atlas.
a Quality control metrics and tissue composition across patients and cell type clusters. Violin plots show distributions of the number of genes, total counts, percentage of mitochondrial counts, and percentage of ribosomal counts. Barplots visualize the relative tissue composition and the total number of cells on a log scale. **b** Marker gene expression for the broad T cell annotations.

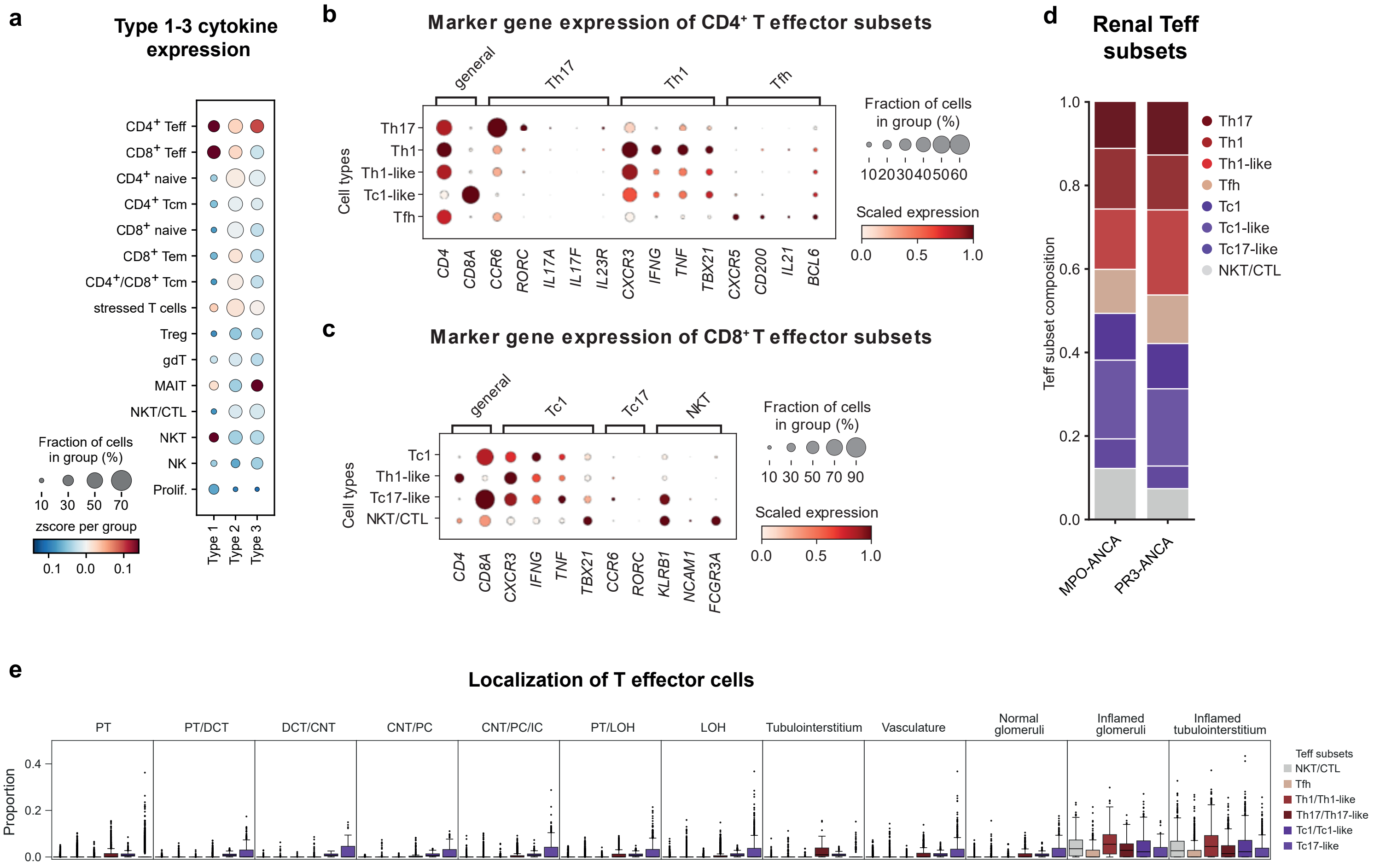

Supplementary Figure 3: Characterization of T effector cells in the exploratory cohort.
a Type 1-3 cytokine scores (type 1: *IFNG, TNF, IL2, IL18, LTA, CSF2*; type 2: *IL4, IL5, IL9, IL13*; type 3: *IL17A, IL17F, IL22, IL26*) for the identified T cell clusters. **b** Marker gene expression for the CD4+ Teff subsets. **c** Marker gene expression for the CD8+ Teff subsets. **d** Comparison of CD4+ and CD8+ T effector cell subsets and their relative proportions between MPO-ANCA and PR3-ANCA positive GN. **e** Distribution of T effector subsets within each renal compartment. Boxplots show the median (middle horizontal line), interquartile range (box), Tukey-style whiskers (lines beyond the box), outliers (data points beyond 1.5*interquartile or below -1.5*interquartile) for proportion of Teff subsets in 10,763 spots from all ANCA ST slides.
PT, proximal tubules. DCT, distal convoluted tubules. CNT, connecting tubules. PC, principal cells. IC, intercalated cells. LOH, loop of Henle.

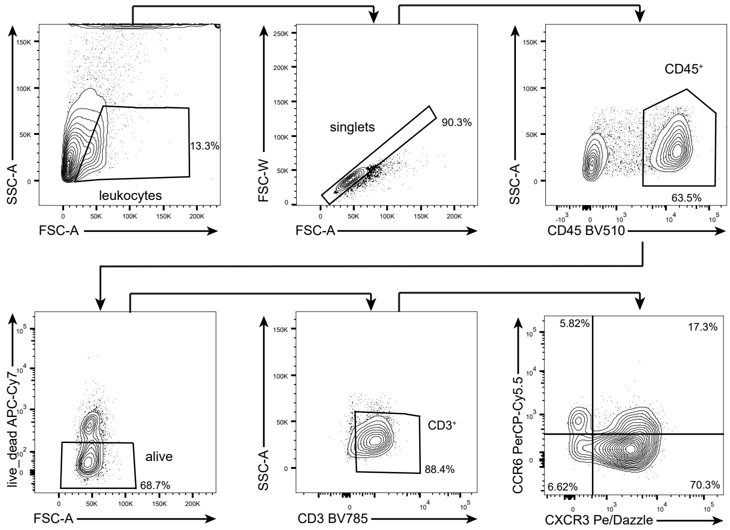

Supplementary Figure 4: Gating strategy rapid biopsy immune profiling. Gating strategy for the identification of the chemokine receptors CXCR3 (Th1/Tc1) and CCR6 (Th17/Tc17) of renal $CD45^+CD3^+$ T cells used in Figure 4 and Supplemental Figure 6. Bottom right panel coressponding to Figure 4a

a

Patient 1

Case

A 73 year old male patient with a history of MPO-ANCA positive vasculitis was referred to a regional hospital in august 2020 with an episode of macro-hematuria, as well as fatigue, dizziness and progressive lower limb edema. Laboratory assessment showed acute kidney injury and elevated CRP levels. Chest imaging excluded relevant pathologies. Urinary analysis showed gross hematuria and macro-albuminuria. BVAS was elevated to 9 points. Suspecting a relapse of AAV, the patient was transferred to university hospital Hamburg-Eppendorf.

AAV history

Initial diagnosis of AAV was made in 2017, with general symptoms, skin and kidney involvement. Kidney biopsy showed pauci-immune crescentic glomerulonephritis with small amounts of mesangial IgA deposition. Induction treatment consisted of PLEX, steroids and cyclophosphamide (cumulative CYC dose 5.0 g). Azathioprine was given for remission maintenance. After 7 months, azathioprine was discontinued due to severe side effects, and rituximab was started. Rituximab was well tolerated, and stable remission achieved, although full B cell depletion was not achieved during maintenance therapy with rituximab. Last dose RTX was given 6 months before re-admission with suspected AAV relapse.

Diagnostic workup at relapse

Hemoglobin [g/dl]	12.0
Leukocytes [10^9/l]	7.8
Platelets [10^9/l]	271
Creatinie [mg/dl]	4.25
eGFR [ml/min]	13
CRP [mg/l]	36
MPO-ANCA [U/ml]	22
B cells [%]	6 (34/µl)

Kidney biopsy was performed, displaying a relapse of ANCA-GN with a high ARRS. Rapid biopsy immune profiling of the kidney biopsy revealed an enrichment of Th1/Tc1 an Th17/Tc17 cells in the kidney tissue.

Treatment considerations

Patient tolerated rituximab well, but full B cell depletion could not be achieved, despite appropriate RTX-dosage. Furthermore, the patient suffered a relapse while on RTX treatment. Urinary flow obstruction and the need of urinary diversion led the patient away from full dose CYC, while still giving consent to two doses of i.v. CYC in the inpatient setting. Rapid immune profiling showed an increase in Th1/Tc1 and Th17/Tc17 cells, suggesting suitabiltty for an anti-IL12/IL-23 treatment.

Treatment

- Pulsed i.v. steroids (3 x 500 mg prednisolone), followed by an oral tapering, according to the PEXIVAS trial reduced dose regimen. By 6 months prednisolone was tapered to 5mg daily (cumulative prednisolone dose: 3900 mg).
- Two i.v. pulses of cyclophosphamide (cumulative dosage 2g) were given in the inpatient setting.

Outcome

Combined treatment of ustekinumab, steroids and low dose CYC resulted in a rapid clinical improvement with resolution of fatigue, edema and dizziness. Creatinine rapidly improved and uACR declined, as well as ANCA-levels, CRP, and BVAS. At 6 months the patient had an elevated BVAS of 3 because of grade 1 hypertension and persistent albuminuria, mostly attributed to glomerular scarring.

organ involvement

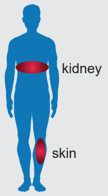

medical history
- monoclonal B cell lymphocytosis
- exogen-allergic asthma
- OSA
- obesity

b

Patient 2

Case

A 52 year old male patient with known history of MPO-ANCA positive vasculitis with extensive organ manifestation was admitted to university medicine hospital Hamburg-Eppendorf, because of increasing creatinine and albuminuria after induction treatment with steroids (3 x 500 mg prednisolone and oral taper) and six pulses of i.v. cyclophosphamide (cumulative dose of 6.0 g; last dose given 4 weeks prior to admission). Initial manifestations (ENT, pulmonary, nervous and cutaneous) had improved since starting of induction treatment with steroids and CYC. BVAS was increased to 12 at admission. With suspected worsening ANCA-GN kidney biopsy was initiated.

AAV history

Initial diagnosis of AAV was made 02/2021 with general, renal, ENT, lung, nervous system, cutaneous, renal and heart manifestation. Treatment with i.v. prednisolone followed by an oral taper (according to the PEXIVAS reduced dose regimen), as well as pulsed i.v. cyclophosphamide (according to EUVAS scheme, cumulative dose 6.0 g) was initiated.

Diagnostic workup at relapse

Hemoglobin [g/dl]	10.7
Leukocytes [10^9/l]	6.5
Platelets [10^9/l]	282
Creatinine [mg/dl]	2.27
eGFR [ml/min]	32
CRP [mg/l]	5
MPO-ANCA [U/ml]	29

Urinary sediment showed dysmorphic erythrocytes (acanthocytes). Kidney biopsy demonstrated pauci-immune crescentic glomerulonephritis with chronic and active lesions, compatible with active ANCA-GN with a low ARRS. Rapid immune-profiling of the kidney biopsy revealed an enrichment of Th1/Tc1 and Th17/Tc17 cells in the kidney tissue.

Treatment considerations

Cyclophosphamide treatment along with steroids could not achieve remission and renal manifestation showed a progressive disease with active glomerular lesions. COVID-19 pandemic was still on the rise, raising concerns over rituximab treatment. Rapid immune profiling of the kidney showed an enrichment of Th1/Tc1 and Th17/Tc17 cells.

Treatment

- Pulsed steroids (3x500mg prednisolone), followed by an oral tapering. By 6 months prednisolone was tapered to 5mg daily (cumulative prednisolone dose: 3755 mg).
- Two i.v. pulses of cyclophosphamide (cumulative dosage 2.0 g) were given.
- Ustekinumab 90mg s.c. was administered at weeks 0, 4, 12, and 24.
- Azathioprine (1 mg/kg) was started at week 22 after beginning of induction therapy as additional maintenance therapy.

Outcome

Combined treatment of ustekinumab, steroids and low dose CYC resulted in a rapid clinical improvement with reduction in creatinine, uACR and ANCA-levels (16 U/ml). At 6 months the patient had an elevated BVAS of 2, because of persistent microalbuminuria, undulating around 200 mg/day.

organ involvement

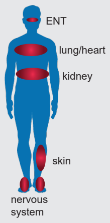

medical history
- hyperlipidemia
- postherpetic neuralgia

c

Patient 3

Case

A 32 year old male with a history of PR3-ANCA positive vasculitis was re-admitted to university hospital Hamburg-Eppendorf, because of fewer, night sweats, weight loss, and progressive dyspnea with hemoptysis. Four weeks before admission the patient was diagnosed with an AAV relapse- rituximab (two pulses of 1g) and pulsed steroids as well as an oral tapering regimen were started. Clinical examination at re-admission showed moist crackles in chest auscultation. BVAS was 13 at that time.

AAV history

Initial diagnosis of AAV was made 2019 and treatment with i.v. prednisolone followed by an oral taper (according to the PEXIVAS reduced dose regimen) as well as rituximab was initiated. After two doses (1g each) of rituximab, the patient was lost to follow up. In 09/2022 aforementioned relapse with pulmonary and ENT manifestation occurred and pulsed prednisolone as well as re-induction with rituximab (two times 1g) was started. Initially the patient's symptoms improved and he was discharged.

Diagnostic workup at relapse

Hemoglobin [g/dl]	8.2
Leukocytes [10^9/l]	1.7
Platelets [10^9/l]	435
Creatinie [mg/dl]	1.88
eGFR [ml/min]	46
CRP [mg/l]	190
PR3-ANCA [U/ml]	101
B cells [%]	0 (1/µl)

CT scans and bronchoscopy showed progressive DAH. Urinary sediment showed extensive acanthocyturia. Kidney biopsy was performed and displayed pauci-immune crescentic glomerulonephritis with active lesions, compatible with ANCA-GN, with a low ARRS. Rapid immune-profiling of the kidney biopsy revealed an enrichment of Th1/Tc1 and Th17/Tc17 cells in the kidney tissue.

Treatment considerations

Severe leukopenia contradicted full dose cyclophosphamide re-induction treatment. Patient was also concerned of cyclophosphamide treatment-related adverse effects, and initially rejected an i.v. treatment. B cells were fully depleted, as rituximab was given four weeks prior. Rapid immune profiling of the kidney showed an enrichment of Th1/Tc1 and Th17/Tc17 cells.

Treatment

- Pulsed steroids (3x250mg prednisolone), followed by an oral tapering. By 6 months prednisolone was tapered to 5mg daily (cumulative prednisolone doses 3520 mg).
- Oral cyclophosphamide was given as the patient was initially rejecting i.v. therapy. After repeated shared decision talks, the patient gave consent to one more i.v pulse under close monitoring of WBC (cumulative dose: 500 mg p.o. and 1000mg i.v.).
- Ustekinumab 90mg s.c. was administered at weeks 0, 4, 12, and 24.
- Starting at week 16 after re-induction therapy AZA was started for additional maintenance therapy and eventually switched to low dose MMF (2x500 mg) because of AZA side effects.

Outcome

Combined treatment of ustekinumab, steroids and low dose CYC resulted in a rapid clinical improvement with suspended night sweats, fever and hemoptysis, a reduction in creatinine, ANCA-levels, and BVAS. At 6 months the patient had an elevated BVAS of 2, because of persisting albuminuria. Antiproteinuric treatment with ACEi was not well tolerated and could only be given at the lowest dose.

organ involvement

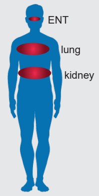

medical history
- no chronic diseases

d

Patient 4

Case

A 72 year old female with a history of MPO-ANCA positive vasculitis (renal limited disease) and myelodysplastic syndrome was admitted to university medicine hospital Hamburg-Eppendorf, because of acute kidney injury and reduced general condition. The patient was receiving rituximab as remission maintenance, with the last dose given 5 months prior to hospital admission. BVAS was 13 at admission. With suspected relapse of ANCA-GN diagnostic workup was initiated.

AAV history

Initial diagnosis of AAV was made in august 2020 with a renal limited phenotype. Treatment with i.v. prednisolone followed by an oral taper (according to the PEXIVAS reduced dose regimen) as well as pulsed i.v. cyclophosphamide (cumulative dose 3600 mg) was initiated. After remission induction azathioprine was given for remission maintenance. Eventually, azathioprine had to be discontinued because of side effects and rituximab at doses of 500 mg/6 months was initiated in 05/2022.

Diagnostic workup at relapse

Hemoglobin [g/dl]	8.9
Leukocytes [10^9/l]	3.0
Platelets [10^9/l]	264
Creatinine [mg/dl]	3.42
eGFR [ml/min]	13
CRP [mg/l]	28
MPO-ANCA [U/ml]	>134
B cells [%]	2 (20/µl)

Chest X-ray showed no signs of pulmonary involvement. Urinary analysis showed extensive acanthocyturia and macro-albuminuria. Kidney biopsy was performed and showed pauci-immune crescentic glomerulonephritis with active lesions, compatible with ANCA-GN, with a high ARRS. Rapid immune-profiling of the kidney biopsy revealed an enrichment of Th1/Tc1 and Th17/Tc17 cells in the kidney tissue.

Treatment considerations

Leukopenia of 3.0 $x10^9$/l with known myelodysplastic syndrome under high dose erythropoietin therapy contradicted full dose cyclophosphamide re-induction therapy. Retrospective the patient showed a rise in MPO-ANCAs in 01/2023 while B cells were depleted (0% / 1/µl). Rapid immune profiling of the kidney showed an enrichment of Th1/Tc1 and Th17/Tc17 cells.

Treatment

- Pulsed steroids (3 x 500 mg prednisolone), followed by an oral tapering regimen according to the PEXIVAS study reduced dose arm. By 6 months prednisolone was tapered to 5mg daily (cumulative prednisolone dose: 4285 mg).
- Three pulses of i.v. cyclophosphamide were given (cumulative dose of 1800mg).
- Ustekinumab 90mg s.c. was administered at weeks 0, 4, 12, and 24.
- Low dose MMF (2 x 500 mg) was stared at week 16 after beginning of induction therapy as additional maintenance therapy.

Outcome

Combined treatment of ustekinumab, steroids and low dose CYC resulted in a rapid clinical improvement with substantial improvement of general condition, reduction in creatinine, uACR, ANCA-levels, and BVAS reduction to 5. At 6 months the patient had an elevated BVAS of 5, because of albuminuria and eumorphic hematuria.

organ involvement

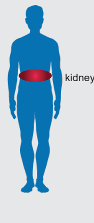

medical history
- myelodysplastic syndrome
- osteoporosis
- Hashimoto's thyroiditis

Supplementary Figure 5: Detailed case vignettes for each patient of the ustekinumab treatment cohort.
Case vignettes illustrating a brief case description, history, diagnostic workup, as well as treatment considerations, treatment and outcomes for each patient of the ustekinumab treatment cohort. **a** Patient 1. **b** Patient 2. **c** Patient 3. **d** Patient 4. BVAS, Birmingham Vasculitis Activity Score. AAV, ANCA-associated vasculitis. PLEX, therapeutic plasma exchange. CYC, cyclophosphamide. ARRS, ANCA renal risk score (21). RTX, rituximab. uACR, urinary albumin to creatinine ratio. OSA, obstructive sleep apnoea. ENT, ear nose throat. DAH, diffuse alveolar hemorrhage. MMF, mycophenolate mofetil. ACEi, angiotensin-converting enzyme inhibitor.

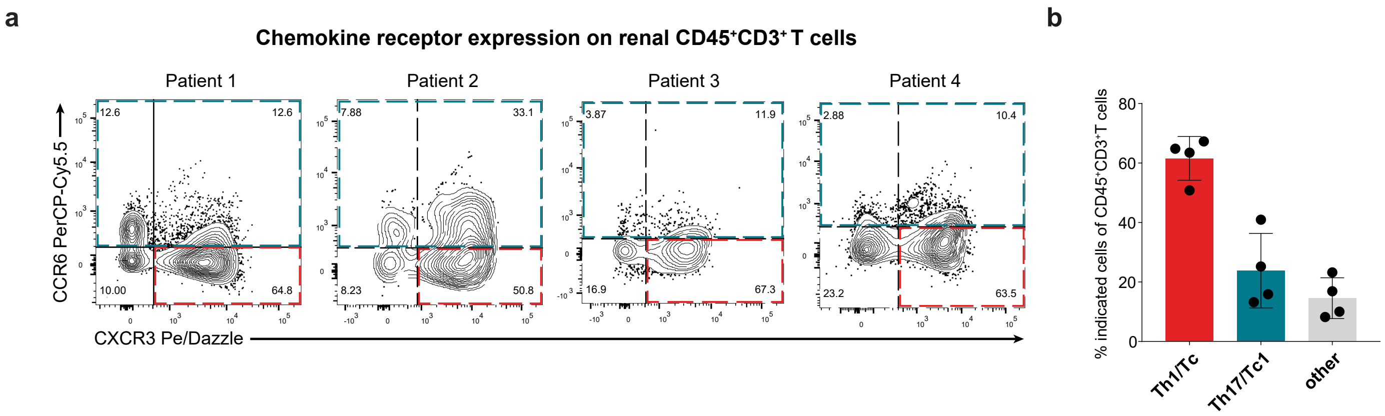

Supplementary Figure 6: Rapid Immunoprofiling of T cells in the kidneys of the treatment cohort.
a Flow cytometry-based identification of chemokine receptor expression from T cells isolated from biopsy samples of patients with ANCA-GN (n=4). **b** Quantification of chemokine receptor expression CXCR3 (Th1/Tc1) and CCR6 (Th17/Tc17) from renal $CD45^{+}CD3^{+}$T cells. Bar graphs show mean with SD, symbols represent individual data points.

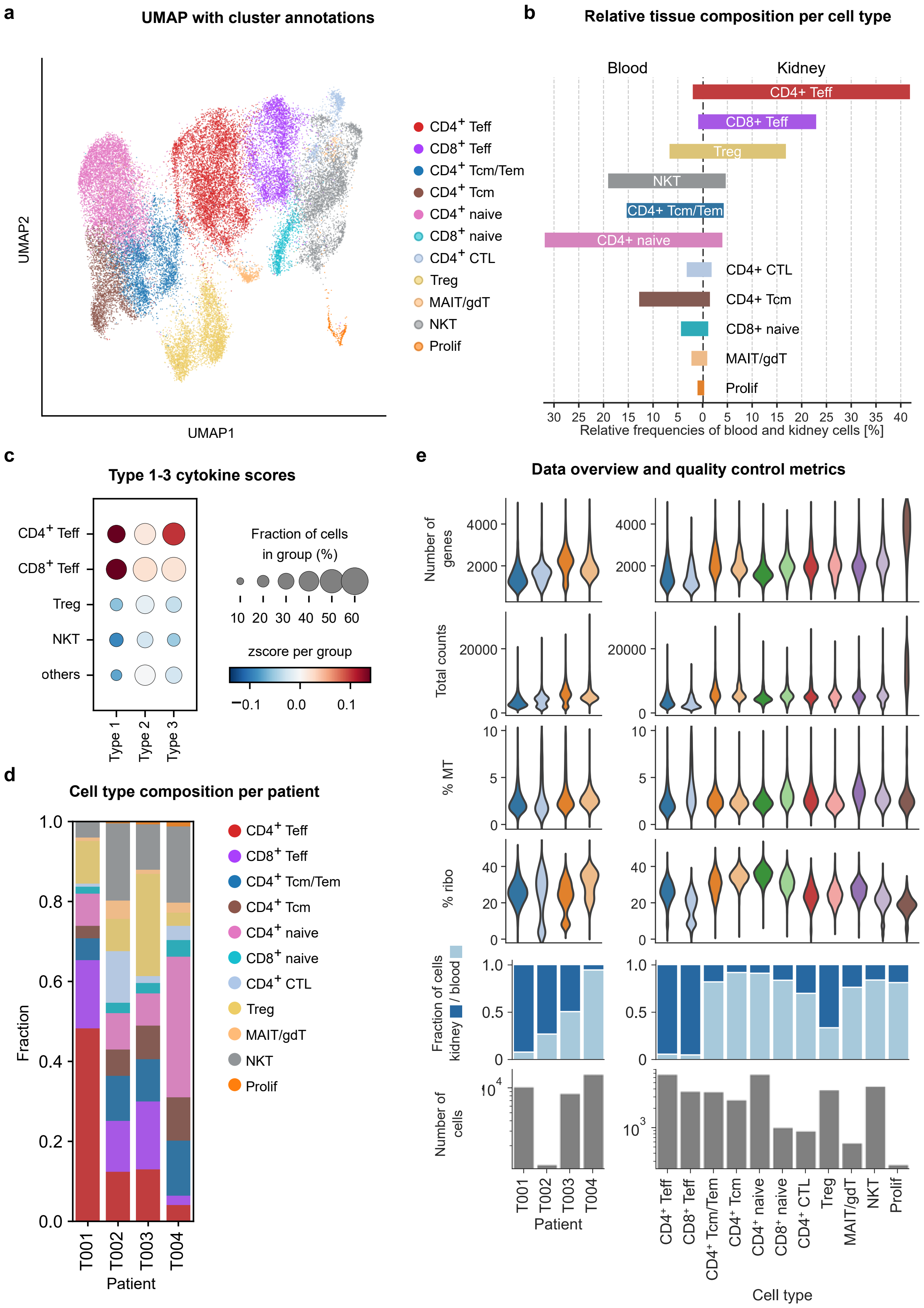

Supplementary Figure 7: Ustekinumab treatment group single-cell T cell atlas.
a UMAP projection of the integrated single-cell embeddings with corresponding cluster annotations. **b** Tissue composition for the different T cell clusters ordered by descending kidney enrichment. **c** Type 1-3 cytokine scores (type 1: *IFNG, TNF, IL2, IL18, LTA, CSF2*; type 2: *IL4, IL5, IL9, IL13*; type 3: *IL17A, IL17F, IL22, IL26*) for the T cell clusters most enriched in the kidney and the other clusters combined. **d** Cell type composition per patient. **e** Quality control metrics and tissue composition across patients and cell type clusters. Violin plots show distributions of the number of genes, total counts, percentage of mitochondrial counts, and percentage of ribosomal counts. Barplots visualize the relative tissue composition and the total number of cells on a log scale.

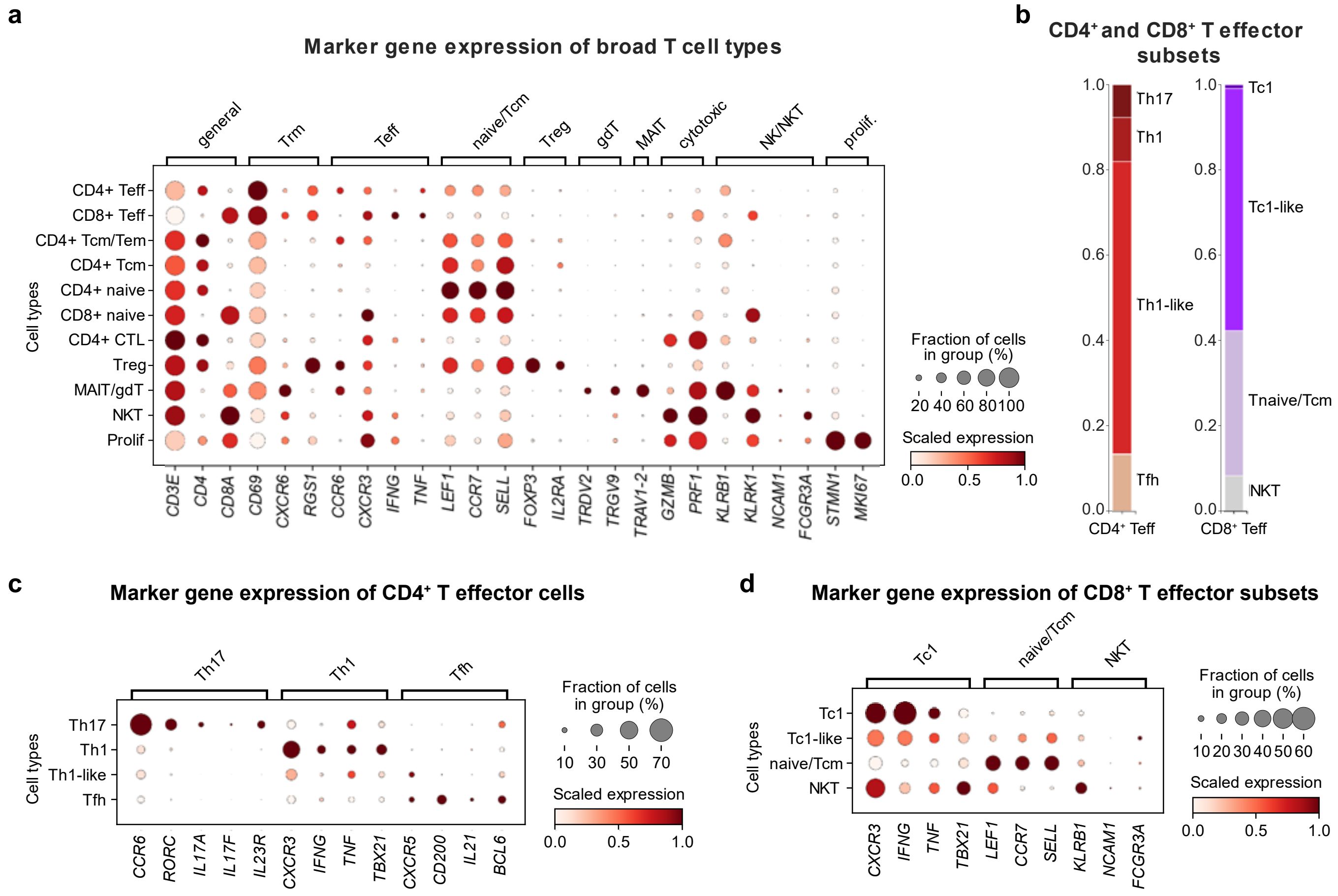

Supplementary Figure 8: Ustekinumab treatment group single cell T cell atlas marker genes and T effector cell type composition. **a** Marker gene expression for the broadly annotated T cell clusters. **b** CD4+ and CD8+ T effector subset cell type composition. **c** Marker gene expression for the CD4+ Teff subsets. **d** Marker gene expression for the CD8+ Teff subsets.
