# Supplementary material for: Immune profiling-based targeting of pathogenic T cells with ustekinumab in ANCA-associated glomerulonephritis
Source: Nat Commun. 2024 Sep 19;15:8220. doi: 10.1038/s41467-024-52525-w (PMC11413367; doi:10.1038/s41467-024-52525-w)
Supplement: Supplementary file 3 — Description of Additional Supplementary Files [file 41467_2024_52525_MOESM3_ESM.pdf]

Description of Additional Supplementary Files

Supplementary Data 1: Alignment metrics of ST data from the ANCA-GN exploratory group.

Supplementary Data 2: Summary of differential population analysis in the ST data between the control samples and the ANCA-GN exploratory group. The analysis was performed using the standard scCODA model with CNT/PC cluster as the reference cell type.

Supplementary Data 3: Differential gene expression of the renal compartments in the ST data from the ANCA-GN exploratory group. Differential gene expression was performed using two-sided Wilcoxon rank-sum tests with Benjamini-Hochberg multiple test correction.

Supplementary Data 4: Gene set enrichment analysis of the inflamed interstitial compartment in the ANCA-GN exploratory group. Enrichment was performed using clusterProfiler enrichGO function that used right-tailed Fisher's Exact test with Benjamini-Hochberg multiple test correction. Statistical significance was tested using the *enrichGO* function from R package clusterProfiler with right-tailed Fisher's exact test and Benjamini-Hochberg multiple test correction.

Supplementary Data 5: Gene set enrichment analysis of the inflamed glomerular compartment in the ANCA-GN exploratory group. Statistical significance was tested using the *enrichGO* function from R package clusterProfiler with right-tailed Fisher's exact test and Benjamini-Hochberg multiple test correction.

Supplementary Data 6: Post QC quality metrics for the single cell sequencing data of the exploratory cohort.

Supplementary Data 7: Final drug candidates and their scores in ANCA-GN exploratory group and Teff cells from the ANCA-GN T cell atlas. Statistical significance was tested using the *enrichGO* function from R package clusterProfiler with right-tailed Fisher's exact test and Benjamini-Hochberg multiple test correction.

Supplementary Data 8: Post QC quality metrics for the single cell sequencing data of the ustekinumab treatment cohort.

Supplementary Data 9: Alignment metrics of the ST data from the internal control group.

Supplementary Data 10: Data overview including publication status for the CITE-/scRNA-seq datasets per patient and tissue.
Detailed information about performed analysis of each patient, as well as information about the immunosuppressive treatment up to 7 days prior to kidney biopsy. Furthermore, all accession codes to the gene expression data listed.
(B, blood: k, kidney; MPO, myeloperoxidase; PR3, proteinase 3; RTX, rituximab; CYC, cyclophosphamide; AZA, azathioprine)
