# Supplementary material for: Immune profiling-based targeting of pathogenic T cells with ustekinumab in ANCA-associated glomerulonephritis
Source: Nat Commun. 2024 Sep 19;15:8220. doi: 10.1038/s41467-024-52525-w (PMC11413367; doi:10.1038/s41467-024-52525-w)
Supplement: Supplementary file 14 — Reporting Summary [file 41467_2024_52525_MOESM14_ESM.pdf]

nature portfolio

Corresponding author(s): Stefan Bonn, Christian F. Krebs, and Ulf, Panzer

Last updated by author(s): Aug 6, 2024

Reporting Summary

Nature Portfolio wishes to improve the reproducibility of the work that we publish. This form provides structure for consistency and transparency in reporting. For further information on Nature Portfolio policies, see our Editorial Policies and the Editorial Policy Checklist.

Statistics

For all statistical analyses, confirm that the following items are present in the figure legend, table legend, main text, or Methods section.

n/a	Confirmed	
☐	☒	The exact sample size (n) for each experimental group/condition, given as a discrete number and unit of measurement
☐	☒	A statement on whether measurements were taken from distinct samples or whether the same sample was measured repeatedly
☐	☒	The statistical test(s) used AND whether they are one- or two-sided<br>*Only common tests should be described solely by name; describe more complex techniques in the Methods section.*
☐	☒	A description of all covariates tested
☐	☒	A description of any assumptions or corrections, such as tests of normality and adjustment for multiple comparisons
☐	☒	A full description of the statistical parameters including central tendency (e.g. means) or other basic estimates (e.g. regression coefficient) AND variation (e.g. standard deviation) or associated estimates of uncertainty (e.g. confidence intervals)
☐	☒	For null hypothesis testing, the test statistic (e.g. F, t, r) with confidence intervals, effect sizes, degrees of freedom and P value noted<br>*Give P values as exact values whenever suitable.*
☒	☐	For Bayesian analysis, information on the choice of priors and Markov chain Monte Carlo settings
☒	☐	For hierarchical and complex designs, identification of the appropriate level for tests and full reporting of outcomes
☒	☐	Estimates of effect sizes (e.g. Cohen's d, Pearson's r), indicating how they were calculated

Our web collection on statistics for biologists contains articles on many of the points above.

Software and code

Policy information about availability of computer code

Data collection	All data were uploaded to the GEO repository (GSE200880, GSE253633, GSE270533, and GSE250138)
Data analysis	The code to process and analyze the single cell sequencing and ST data is available at https://github.com/imsb-uke/ANCA-GN_transcriptomics.<br><br>DOI: https://doi.org/10.5281/zenodo.13208437.

For manuscripts utilizing custom algorithms or software that are central to the research but not yet described in published literature, software must be made available to editors and reviewers. We strongly encourage code deposition in a community repository (e.g. GitHub). See the Nature Portfolio guidelines for submitting code & software for further information.

Data

Policy information about availability of data

All manuscripts must include a data availability statement. This statement should provide the following information, where applicable:

- Accession codes, unique identifiers, or web links for publicly available datasets
- A description of any restrictions on data availability
- For clinical datasets or third party data, please ensure that the statement adheres to our policy

All gene expression data used in this manuscript are publicly available via the NCBI Gene Expression Omnibus (https://www.ncbi.nlm.nih.gov/geo/). The newly generated data for this study is accessible under GSE253633 (https://www.ncbi.nlm.nih.gov/geo/query/acc.cgi?&acc=GSE253633) and GSE250138 (https://

www.ncbi.nlm.nih.gov/geo/query/acc.cgi?acc=GSE250138). The accession codes for all other gene expression data used are listed in the Supplementary Data 10.

Research involving human participants, their data, or biological material

Policy information about studies with human participants or human data. See also policy information about sex, gender (identity/presentation), and sexual orientation and race, ethnicity and racism.

Reporting on sex and gender	Due to the sample size, sex- and gender-based analyses were not performed. Information about the sex of the the patients is provided in Table 1 for the exploratory cohort and in Table 2 for the treatment cohort. Self reported sex and biological sex were identical in the exploratory and treatment cohort.
Reporting on race, ethnicity, or other socially relevant groupings	Data on race, ethnicity or other socially relevant groupings were not included.
Population characteristics	Information about the population characteristics is provided in Table 1 for the exploratory cohort and in Table 2 for the treatment cohort.
Recruitment	All patients recruited were included in the Hamburg GN Registry. All patients provided informed consent. Patients were enrolled between June 2017 and April 2023. Follow up data was collected until Oktober 2023.
Ethics oversight	Ethik-Kommission der Ärztekammer Hamburg, (local ethics committee of the chamber of physicians in Hamburg)

Note that full information on the approval of the study protocol must also be provided in the manuscript.

Field-specific reporting

Please select the one below that is the best fit for your research. If you are not sure, read the appropriate sections before making your selection.

[x] Life sciences [ ] Behavioural & social sciences [ ] Ecological, evolutionary & environmental sciences

For a reference copy of the document with all sections, see nature.com/documents/nr-reporting-summary-flat.pdf

Life sciences study design

All studies must disclose on these points even when the disclosure is negative.

Sample size	Exploratory cohort: n=34; Treatment cohort: n=4.
Data exclusions	In the spatial transcriptomics data analysis V4_A was excluded from the analysis as both the median gene- and UMI-counts were different from the other slides.
Replication	Throughout the 38 samples being processed, that data was largely consistent. Comparison between the samples is shown in Figure 4 (flow cytometry - exploratory cohort), Supplemental Figure 6 (flow cytometry - treatment cohort) Supplementary Figures 1,2 (spatial transcriptomics and scRNA-seq - exploratory cohort) and Supplementary Figures 7,8 (spatial transcriptomics and scRNA-seq - treatment cohort)
Randomization	No randomization was performed.
Blinding	No blinding was performed.

Reporting for specific materials, systems and methods

We require information from authors about some types of materials, experimental systems and methods used in many studies. Here, indicate whether each material, system or method listed is relevant to your study. If you are not sure if a list item applies to your research, read the appropriate section before selecting a response.

Materials & experimental systems

n/a	Involved in the study	
[ ]	[x]	Antibodies
[x]	[ ]	Eukaryotic cell lines
[x]	[ ]	Palaeontology and archaeology
[x]	[ ]	Animals and other organisms
[ ]	[x]	Clinical data
[x]	[ ]	Dual use research of concern
[x]	[ ]	Plants

Methods

n/a	Involved in the study	
[x]	[ ]	ChIP-seq
[ ]	[x]	Flow cytometry
[x]	[ ]	MRI-based neuroimaging

Antibodies

Antibodies used	Flow cytometry/FACS:<br>Cells were stained with fluorochrome-conjugated antibodies from BioLegend and BD Biosciences, CD45 BV510 (BioLegend, clone HI30, catalogue number 304036, dilution 1:100), CD3 BV785 (BioLegend, clone OKT3, catalogue number 317330, dilution 1:200), CD4 BV650 (BioLegend, clone RPA-T4, catalogue number 300536, dilution 1:200), CD8 APC-R700 (BD Bioscoences, clone RPA-T8, catalogue number 565165, dilution 1:100), CXCR3 Pe/Dazzle (BioLegend, clone G025H7, catalogue number 353736, dilution 1:100), CCR6 PerCP-Cy5-5 (BioLegend, clone G034E3, catalogue number 353406, dilution 1:100).<br><br>Immunofluorescence:<br>CD3 (Abcam, ab11089, dilution 1:100), CCR6 (Sigma, HPA014488/Origene TA316610, dilution 1:100), and CXCR3 (BD Biosciences, 557183, dilution 1:100)
Validation	Flow cytometry/FACS<br>https://www.biolegend.com/de-de/products/brilliant-violet-510-anti-human-cd45-antibody-8006<br>https://www.biolegend.com/en-us/products/brilliant-violet-785-anti-human-cd3-antibody-7977<br>https://www.biolegend.com/en-us/products/brilliant-violet-650-anti-human-cd4-antibody-7650<br>https://www.bdbiosciences.com/en-us/products/reagents/flow-cytometry-reagents/research-reagents/single-color-antibodies-ruo/apc-r700-mouse-anti-human-cd8.565165<br>https://www.biolegend.com/en-us/products/pe-dazzle-594-anti-human-cd183-cxcr3-antibody-10363<br>https://www.biolegend.com/en-us/products/percp-cyanine5-5-anti-human-cd196-ccr6-antibody-7559<br><br>Immunoflurescence:<br>https://www.abcam.com/en-us/products/primary-antibodies/cd3-antibody-cd3-12-ab11089#<br>https://www.bdbiosciences.com/en-us/products/reagents/flow-cytometry-reagents/research-reagents/single-color-antibodies-ruo/purified-mouse-anti-human-cd183.557183<br>https://www.sigmaaldrich.com/DE/de/product/sigma/hpa014488

Clinical data

Policy information about clinical studies
All manuscripts should comply with the ICMJEguidelines for publication of clinical research and a completedCONSORT checklist must be included with all submissions.

Clinical trial registration	n/a
Study protocol	n/a
Data collection	Clinical data was obtained from the Hamburg Glomerulonephritis Registry.
Outcomes	n/a

Plants

Seed stocks	*Report on the source of all seed stocks or other plant material used. If applicable, state the seed stock centre and catalogue number. If plant specimens were collected from the field, describe the collection location, date and sampling procedures.*
Novel plant genotypes	*Describe the methods by which all novel plant genotypes were produced. This includes those generated by transgenic approaches, gene editing, chemical/radiation-based mutagenesis and hybridization. For transgenic lines, describe the transformation method, the number of independent lines analyzed and the generation upon which experiments were performed. For gene-edited lines, describe the editor used, the endogenous sequence targeted for editing, the targeting guide RNA sequence (if applicable) and how the editor was applied.*
Authentication	*Describe any authentication procedures for each seed stock used or novel genotype generated. Describe any experiments used to assess the effect of a mutation and, where applicable, how potential secondary effects (e.g. second site T-DNA insertions, mosiacism, off-target gene editing) were examined.*

Flow Cytometry

Plots

Confirm that:

- [x] The axis labels state the marker and fluorochrome used (e.g. CD4-FITC).
- [x] The axis scales are clearly visible. Include numbers along axes only for bottom left plot of group (a 'group' is an analysis of identical markers).
- [x] All plots are contour plots with outliers or pseudocolor plots.
- [x] A numerical value for number of cells or percentage (with statistics) is provided.

Methodology

Sample preparation	Single cell suspensions were obtained from human kidney biopsies by enzymatic digestion in RPMI 1640 medium with collagenase D at 0.4 mg/ml (Roche, 11088858001) and deoxyribonuclease I (DNase I; 10 µg/ml; Sigma-Aldrich, 10104159001) at 37°C for 30 min followed by dissociation with gentle MACS (Miltenyi Biotec). Leukocytes from blood samples were separated using Leucosep tubes (Greiner Bio-One, 10349081). Cells were stained with fluorochrome-conjugated antibodies from BioLegend and BD Biosciences. Cells were also stained with a dead cell stain to exclude dead cells from analysis.
Instrument	FACSAria Fusion cell sorter (BD Biosciences, Heidelberg, Germany)
Software	Flow cytometry data were analyzed with FlowJo v10.9.
Cell population abundance	80% - 95%
Gating strategy	The gating strategy is provided in Extended Data Figure 4a; SSC-A/FSC-A leukocytes -> FSC-W/FSC-A singlets -> SSC-A/CD45 BV510 CD45+ -> live_dead APC-Cy7/FSC-A alive -> SSC-A/CD3 BV785 CD3+ -> CCR6 PerCP Cy5.5/CXCR3 PE Texas Red Th1/Tc1, Th17/Tc17.

☒ Tick this box to confirm that a figure exemplifying the gating strategy is provided in the Supplementary Information.
